# Pathway of Effects of Socioeconomic Status on Rural Left-behind Children to Receive Oral Health Services: A Structural Equation Modeling

**DOI:** 10.3390/ijerph20021068

**Published:** 2023-01-07

**Authors:** Sichen Liu, Virasakdi Chongsuvivatwong, Shinan Zhang, Angkana Thearmontree

**Affiliations:** 1 Improvement of Oral Health Care Research Unit, Community Dentistry Division, Department of Preventive Dentistry, Faculty of Dentistry, Prince of Songkla University, Songkhla 90110, Thailand; 2 Department of Dental Public Health, School of Stomatology, Kunming Medical University, Kunming 650000, China; 3 Department of Epidemiology, Faculty of Medicine, Prince of Songkla University, Songkhla 90110, Thailand

**Keywords:** parental migration, left-behind children, dental care, health care-seeking behavior, health care utilization, structural equation modeling, data visualization, heatmap

## Abstract

In a rural area with a high proportion of left-behind children (LBC), we aimed to identify the pathway of influence of socioeconomic status (SES) on LBCs to receive oral health services after individualized advice. Between September and October 2020, in a rural area of Yunnan, a survey of 238 LBC and 210 non-left-behind children (NLBC) showed that 91.9% and 94.2% had primary teeth caries, respectively. Their caregivers were advised on (1) dental care: bringing the children to seek professional dental care; and (2) self-care: supervising the children’s oral health behaviors. Two to three months later, the children and their caregivers were visited to assess the compliance with these items of advice. Structural equation modeling (SEM) was used to handle the association between SES and compliance with the advice. A heatmap was used to visualize the data of reasons for seeking dental care or not. A total of 183 (87.1%) NLBCs and 206 (86.6%) LBCs were given the above advice; 32.9% of caregivers complied with dental care advice without a statistical difference between the LBC and NLBC group; 69.9% of caregivers of NLBCs complied with self-care advice, statistically more than those LBCs (59.2%). The education of caregivers was significantly associated with compliance with both advice items in univariate analysis. SES had a direct effect on the children being left behind and the level of oral health knowledge and awareness of the caregivers. Being left behind did not have an independent effect on receiving oral health services for children. “Dental disease was not severe” and “having no pain” were the main reasons for not seeking dental care. There was no clear grouping of participants with different background information based on the reasons given for seeking or not seeking dental care. Our study pointed to the importance of SES level. Being left behind alone may not be a risk factor for missing oral health services among rural children.

## 1. Introduction

Addressing the unrealized rights of all vulnerable groups, such as migrant families and left-behind children (LBC), is one of the programs toward achieving the Sustainable Development Goals (SDGs) in China [1]. LBCs refers to children with one or both of their parents having left their home or hometown for work for at least 6 months [2]. Internal migration has stimulated rapid economic growth in China but has also negatively impacted LBCs’ access to basic healthcare services, such as incomplete vaccination [3] and poor nutrition [4]. Consequently, it affects their development and welfare. In China, there were an estimated 69 million LBCs in 2015, accounting for 25% of the entire population of children [5]. About 59% of them lived in rural areas, which meant 3 out of 10 children in rural areas were LBCs [5].

In China, there is a low rate of utilization of oral healthcare services. About 73.4% [6] and 48.7% [7] of rural Chinese children aged 5 and 12 have deciduous and permanent teeth caries. However, the low rates of oral health service utilization correspond to this high prevalence of dental caries. The respective rates of oral health service utilization were 19.2% and 26.4% among 5 and 12-year-old children in the past 12 months, according to the 4th National Oral Health Survey report [8]. Those children living in rural areas would have a relatively lower rate of oral health service utilization than the urban group.

LBCs suffer more oral health problems and economic burden of oral diseases, owing to poor household income and mostly living in rural areas. It has been shown that LBCs have a high prevalence of health and oral health problems, especially dental caries [9,10]. A report on overall health service utilization found that 8.0% of LBCs under 5 years old had hospitalization in the past 12 months [11], and 7.2% of LBCs should have been hospitalized but were without hospitalization [11]. That report afforded a glimpse of the low utilization of healthcare among the LBC group. There is a need for more data on healthcare utilization to help understand LBCs’ situation.

Studies have shown that healthcare utilization for children among low-socioeconomic-status (SES) families is lower than among high-SES families. In South Africa, children from low-SES families have lower immunization coverage [12]. Brazilian preschool children of low SES are less likely to access the oral health service and receive oral prevention than those of high SES [13]. A national oral health survey in Belgium found that 12.6% of children (mean age of 11 years) in low-household-income families had not had a dental visit in the last 5 years [14].

Barriers in the health setting and system and financial difficulty were the known obstacles to available, accessible, and affordable healthcare services for the rural population [15]. The same three obstacles are barriers to oral health services for the rural population in China. First, primary healthcare centers are the most convenient and accessible health services for the rural population. However, they mainly provide general physician services and do not support oral healthcare services [16]. On the other hand, although over 98% of the rural population was covered by national basic medical insurance [17], more than 90% of dental care fees were paid out of pocket [8,17]. Third, the average dental cost in the past 12 months was CNY 311.08 for rural Chinese children [8]. It is unaffordable for children from poverty families, whose average annual income is CNY 2300 [18]. Besides these barriers, we expected that LBCs would be more likely to be neglected for oral health services due to their parents’ absence.

Most studies in the past have examined the children and assessed their service utilization before the survey [8,13,14]. The survey is usually followed by dental health advice to the children and the caregivers, but there has not been any follow-up study to check whether the caregivers changed their behaviors after receiving the advice, including whether they brought the children to see a dentist. Therefore, we were interested in evaluating the response to the dentist’s advice after having the oral examination of their children among the caregivers of both LBCs and non-left-behind children (NLBC).

In September 2020, we conducted an oral health survey in three rural counties of Yunnan where LBCs were common. We found about 91.9% and 19.8% of children with parental migration longer than 12 months had primary and permanent teeth caries, respectively, as well as 94.2% and 30.9% among children without parental migration [10]. After the survey, we gave the caregivers careful tailored feedback and we used the WHO’s [19] recommended procedure to advise them to take their children to visit the dentist to receive further treatment. Subsequently, between December 2020 and January 2021, we conducted a second survey to check caregivers’ compliance with the advice.

This follow-up study aimed to determine whether and how, after receiving individualized dental advice, the compliance to the advice differed between the caregivers of LBCs and those of NLBCs. Other variables in this study were selected based on findings from previous studies, and included socioeconomic variables of the caregivers [8,20] and awareness of dental health [14]. Most previous studies directly predicted oral health care utilization from family SES [8,13,14]. We argue that oral health awareness of the caregivers may be the intermediary variable. The structural equation modeling (SEM), a more advanced form of regression analysis, is an important analytical strategy to disclose the intermediary role of a variable such as awareness [21]. It was used in the study to enhance our understanding of the pathway from SES to the final behavioral outcome. If this intermediary variable is confirmed, and the reasons for noncompliance can be well documented, future interventions should emphasize oral health awareness to improve health care utilization.

The objectives of this study were therefore to (1) compare compliance with dental advice on supervising oral health behaviors and seeking dental care between caregivers of LBCs and NLBCs, (2) identify the pathway of influence of SES on LBCs to receive oral health services after individualized advice, and (3) document the reasons of not complying with dental care advice among caregivers.

## 2. Methods

This study is part of Oral health promotion activities of children in Yunnan, performed by the Department of Dental Public Health, School of Stomatology, Kunming Medical University. The study team consisted mainly of university students with oral health education backgrounds from Kunming Medical University, currently working as interns in the Stomatological Department of the local county hospital. In addition, 6–7 dentists working in the Department of Stomatology of the local county hospitals also participated.

### 2.1. Study Setting and Study Design

Yunnan Province, located in the southwestern corner of China, has a low gross domestic product (GDP), ranking 18 among 31 provinces of China in 2021 [22]. We chose to study in Qujing city, the second-largest prefecture in Yunnan, with the largest number of LBCs in the province [23]. The dentist-population ratio in Qujing was 1:24,809 [24]. There were 91 oral health sectors in Qujing, including 36 public hospitals and 55 private clinics [24]. There is usually an opening time in private dental clinics from 8.30 am to 9.00 pm. Therefore, there were few limitations on the opening time and resources to visit the oral health sectors among children and their caregivers.

The study was conducted from September 2020 to January 2021. The STROBE guidelines were used to ensure the complete reporting of this observational study.

### 2.2. Sample Selection and Sample Size

Out of the nine total counties in Qujing, three rural counties with similar socioeconomic backgrounds were chosen, namely Zhanyi, Huize, and Xuanwei. The 2021 per capita disposable income of rural residents among these counties were USD 2381 [25], USD 1757 [26], and USD 2248 [27], respectively, which were lower than the average GDP of the Yunnan province.

Eighteen rural subdistricts within 1 h traveling time by public transport to local county hospitals were chosen from three counties. Then, seven primary schools with the largest number of LBC were selected from those rural subdistricts. Finally, the children aged 6 to 8 were randomly chosen from these primary schools.

A total of 500 children underwent the oral health examination in the baseline survey. However, the caregivers of 52 children were absent on the survey date, and thus did not receive advice. These participants were removed from the study. Therefore, 448 children and their caregivers participated in this follow-up.

In SEM, the minimum sample size regarding the ratio of the number of cases to the number of model parameters would be 20:1 to be statistically efficient [21]. The minimum sample size would be 180 with 9 parameters, which was based on the present study’s path analysis model (detail in Figure S2). Therefore, a sample of 389 would be efficient for SEM analysis.

### 2.3. Ethics Approval

Ethics approval was given by the Research Ethics Committee (REC), Faculty of Dentistry, Prince of Songkla University (EC6210-038). The Education Bureau of Yunnan Province approval was obtained before the start of the research. Furthermore, all caregivers of children signed informed consent before the survey.

### 2.4. Data Collection

All children finished the oral examination at baseline from September to October 2020. A specific report on the children’s oral health assessment form [19] with the stamp of the Dental Public Health Department was prepared based on the examination results, and included two parts: (1) the results of the child’s oral health assessment—overall assessment of oral health condition, numbers of dental caries; and (2) advice for dental care—cleaning the teeth, the position of extracted and filled teeth, and self-care of their children, such as supervision of tooth brushing in the morning and before bed and reducing the frequency of snacking (details in Figure S1).

This report was handed in to the individual caregivers of the child with explanation on the recommendation, and the following information: the importance of dental care and self-care, detailed location of local dental hospitals/clinics, the mark of fluoride toothpaste, and the familiar brand of children’s toothpaste with fluoride in the local supermarket. Any other questions that caregivers asked were answered. Finally, a kit of children’s toothpaste and toothbrush was given to the caregiver as a gift. Meanwhile, we reminded them to participate in the return event after two or three months.

In December 2020 and January 2021, we revisited those populations to follow up and assess compliance of the caregivers to the advice. A short questionnaire modified from a questionnaire in the national survey of China [6] was completed by caregivers: “According to the last oral examination recommendation, did you followed the advice? yes, or no? and the reasons”. The research assistants read the questions to all illiterate caregivers to help them finish the questions. We conducted phone calls for caregivers who did not come to school to finish the questions. Those who did not answer the phone were regarded as lost to follow-up.

### 2.5. Structural Equation Modeling (SEM) and Variables

SEM is a statistical analysis method to test the hypotheses that many observed variables are in fact part of a latent variable (so-called confirmatory factor analysis) and the relationship of the variables are in cascade with independent, intermediary, and outcome variable(s) [21]. While LBC is the key independent variable, we framed our analysis to have a latent variable reflecting degrees of the child being left behind (type of caregiver and duration of the child being left behind). The other latent variable was SES (education level, occupation, and family belonging of the caregivers), which we considered being a confounding latent variable. We proposed that oral health knowledge and awareness are intermediary variables linked with the final outcome, namely the behavior of bringing a child with dental caries to receive professional dental care (details are in Figure S2). However, other variables related to health of the parents were not available for analysis, and all the study schools were located in the center or subcenter of the county, so the distance from the county was not included in the model.

### 2.6. Data Analysis

Data entry and validation were performed using EpiData 3.0. R software (Version 4.2.2, R Foundation for Statistical Computing, Vienna, Austria) was used to clean and analyze data.

Prior the SEM, chi-square or Fisher’s exact test was used to compare the percentage of characteristics of participants among two follow-up outcomes. Then, three steps of SEM were conducted: preparation of the latent variables of SEM by confirmatory factor analysis (CFA), estimation of the model by pathway analysis, and testing the model’s fitness by several goodness indexes [21].

SEM automatically has all variables standardized (mean becomes zero and standard deviation (SD) becomes the unit). The relationship between two variables was expressed as a coefficient (beta). The beta value should be at least 0.20, and ideally above 0.30, in order to be considered meaningful for discussion [28,29]. Coefficients in SEM were interpreted as the strength of association in the same way as regression coefficients [30]. The ‘Lavaan’ packages in R were used for CFA and SEM. A *p*-value < 0.05 was required for significance.

Data were visualized by quadrant diagrams and heatmaps. Quadrant diagrams are an efficient way of highlighting the distribution of critical demographic factors between the follow-up outcomes. The *x*-axis was the percentage of compliance with self-care advice (to supervise the oral health behaviors of the child), the *y*-axis was the percentage of compliance with dental care advice (to take the child to seek dental care), and the origin was denoted by (50%, 50%). Only quadrant I and IV were displayed based on the data range in the present study.

Cluster heatmap was used to visualize the data on reasons for follow-up outcomes among the participants and reveal similar patterns by cluster. The columns were reasons for seeking or not seeking dental care among participants and are represented in the rows. The hierarchical clustering in the column and row were performed by a hclust object method. The package ‘pheatmap’ in R was used to create the heatmap.

## 3. Results

**Figure 1:**
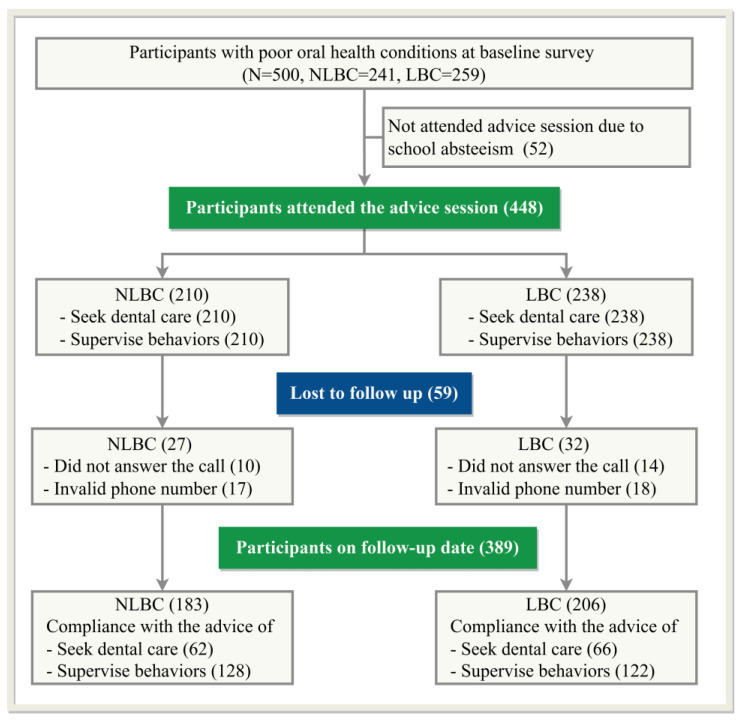
Flow diagram of participation.

As Figure 1 shows, 448 participants received the advice at the baseline survey. However, 59 (13.2%) participants were lost to follow-up, with most of them not answering the call or having invalid phone numbers. Finally, 389 participants with 183 NLBC (47.0%) and 206 LBC (53.0%) completed the follow-up.

### 3.1. Univariate Analysis Results

Table 1 compares the caregivers’ compliance with the advice to supervise children’s oral health behaviors and bring children to seek dental care based on univariate analysis. One-third of caregivers complied with seeking dental care (32.9%). Most of them complied with self-care by supervising the children’s oral health behaviors, but with significantly higher compliance among caregivers of NLBC. Of 15 variables, only education of the caregivers was significantly associated with compliance with both items of advice. Caregivers who were white-collar workers and had appropriate oral health awareness had a higher percentage of compliance with self-care advice. Caregivers of children without siblings had a significantly higher percentage of compliance with seeking dental care.

**Figure 2:**
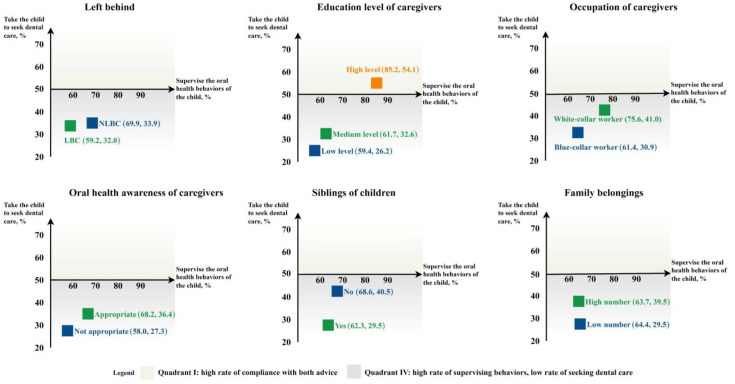
Quadrant diagrams of compliance with supervising oral health behaviors and seeking dental care.

In Figure 2, quadrant I indicates a high percentage of compliance with both items of advice, with more than 50% of them practicing the self-care and dental care advice on their children. Quadrant IV indicates a low percentage of compliance to seek dental care but a high percentage of compliance to supervise oral health behaviors. Six demographic characteristics with a *p*-value less than 0.05 in Table 1 were chosen in these quadrant diagrams. Among the six quadrant diagrams, the outstanding factor was the high education level of caregivers, which was the only one located in quadrant I. It means that caregivers with high education levels are more likely to take their children to seek dental care.

### 3.2. SEM Results

**Figure 3:**
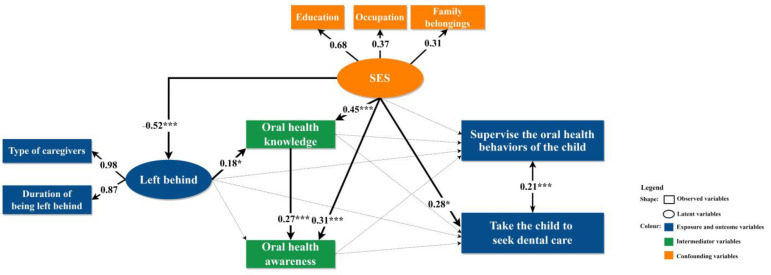
Structural equation modeling of the relationship between being left behind and compliance with advice. Note: The solid lines indicate significant relationships with the number on each line showing standardized path coefficients; the significant level for path coefficients was set at * *p* < 0.05, and *** *p* < 0.001; the dotted lines indicate insignificant relationships.

Figure 3 summarizes the SEM results. Significant coefficients are represented by solid arrows. Being left behind was strongly and negatively associated with SES (β = −0. 52, *p* < 0.001). LBCs tended to have lower SES and their caregivers were more likely to have no formal schooling, be unemployed, and have fewer family belongings compared to NLBCs. High SES was moderately associated with better oral health knowledge (β = 0.45, *p* < 0.001) and awareness (β = 0.31, *p* < 0.001). Finally, the main outcome was that taking the child to seek dental care was weakly associated (β = 0.28, *p* < 0.05) with high SES. Thus, caregivers with high SES were less likely to have LBCs, had more appropriate oral health knowledge and awareness, and had more chances to seek dental care for their children. Being left behind, the main exposure of our study, was not directly associated with the outcomes.

Table S1 summarizes the results of CFA. Two latent variables were constructed in the model: being left behind and SES. Although a low factor loading was shown in the family belongings (factor loading = 0.31), it was kept in SES for reasonable logic. Furthermore, Table S2 summarizes the fitness of the model (SRMR = 0.02, GFI = 0.99). Overall, the model had a good fit.

### 3.3. Reasons for Not Taking Children to Seek Dental Care

**Figure 4:**
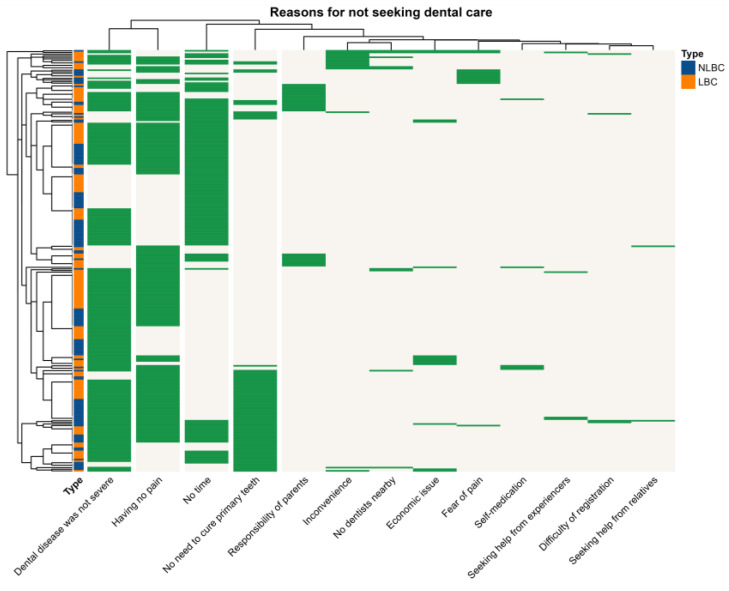
Heatmap showing clustering of reasons for not seeking dental care among participates.

Table 2A summarizes the frequency and percentages of each reason chosen by the caregivers for not bringing the child to see a dentist. The same information is also displayed in Figure 4.

Figure 4 is a heatmap summarizing distribution of reasons for not seeking dental care (in 13 columns) and grouping of the 261 subjects in rows. Columns with similar distribution in rows and rows with similar distribution in columns are automatically placed nearby together by the software.

Thus, the first two columns, ‘dental disease was not severe’ (74.3%) and ‘having no pain’ (62.8%), are close together as they were chosen together by similar sets of participants. Note that both belong to a relatively long branch of the tree, indicating that they are well separated from other reasons. The third reason, ‘no time’, was distinct. It was chosen by approximately half of the participants (51.7%) and by almost none of the remaining half. The remaining reasons chosen by just a few participants were not well grouped, as the length of the trees are relatively short.

Row-wise in Figure 4, the tree has rather similar lengths of branches to groups of subjects. This indicates that there was no clear way of grouping of subjects based on the reason they chose. Note that the strips of NLBC in blue and LBC in brown are distributed throughout all branches. This means that there was no correlation between the reason the participant chose and whether or not they were from LBC families.

### 3.4. Reasons for Taking Children to Seek Dental Care

**Figure 5:**
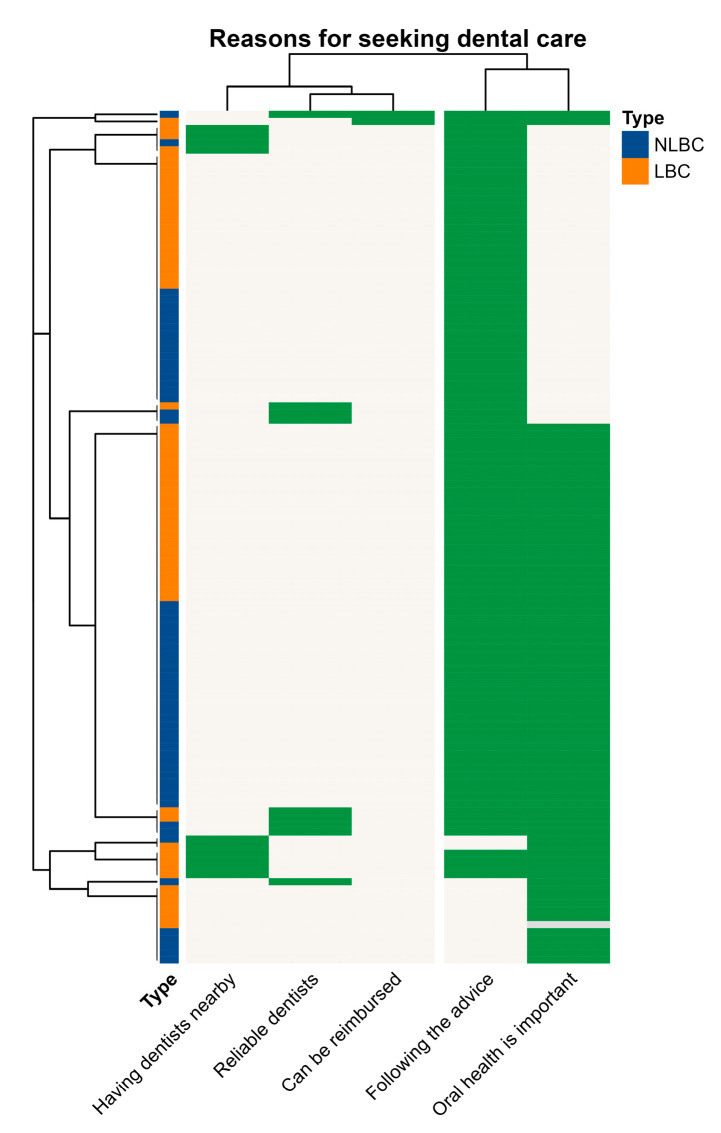
Heatmap showing clustering of reasons for seeking dental care among participates.

In a similar fashion, Table 2B summarizes the numbers and Figure 5 visually displays the reasons for visiting a dentist. The two closest and most common reasons were ‘following the advice’ (88.3%) and ‘oral health is important’ (64.7%). Other reasons were much less common. There was no association with NLBC/LBC status of the children. Supplementary Figures S3–S8 had the strip colored by type, education, and occupation of caregivers. Again, the reasons do not seem to link with their background information.

## 4. Discussion

This study was conducted in a rural population with a high prevalence of long-time rural-urban migration in Yunnan, China. Postsurvey advice was given to caregivers of children with dental caries to give supervision on oral health behaviors and bring the child to see a dentist. A low compliance with dental care advice was found in both LBC and NLBC caregivers. Many participants’ characteristics did not influence compliance with advice, but a high education level of caregivers was strongly associated with better compliance with both items of advice. Caregivers’ SES had the strongest influence on all variables, including children being left behind and compliance with dental care advice. Finally, beliefs that ‘dental disease was not severe’ and ‘having no pain’ were the main reasons for caregivers not to seek the dentist for their child.

This study had a slightly higher proportion of compliance with dental care advice after individualized feedback (32.9%) compared with a similar survey of pupils aged 8 to 11 years (14.7%) [31]. Compared with the study of Onyejaka et al. [31], the present study intervened and received feedback from caregivers rather than children, as caregivers were the decision makers of healthcare utilization for children. Furthermore, it is more effective to motivate subjects using interventions that combine the results of oral health examinations and tailored feedback advice than only performing oral health education. On the other hand, more than two-thirds of caregivers followed the self-care advice, suggesting that the advice we gave strongly motivated those caregivers. It can improve children’s oral health behaviors and hygiene through caregivers’ supervision behaviors, as it mediates between children’s behavior intentions and performed actions [32]. Overall, professional dental advice could motivate rural caregivers to bring the child to seek dental care and practice self-care for their child with poor oral health conditions.

SES of caregivers was the main determining factor for children using oral healthcare services [33]. A Chinese national survey found that children from the family with more than CNY 25,000 annual per capita income were more likely to visit the dentist in the past year [20]. However, the present study was conducted in rural areas, with all three locations having lower incomes than standard. It is possible to explain the lack of statistical significance between caregivers of NLBCs and LBCs in seeking dental care for their child. Although few participants chose ‘economic issues’ as the reason for not seeking dental care, this barrier still exists in the utilization of oral healthcare services among participants. Moreover, it should be noted that this study was conducted during the early period of the COVID-19 pandemic when the population of China was facing difficulties from economic and social problems.

Our study did not find that caregivers taking their children to visit a dentist was directly associated with being left behind, but there was conflicting evidence presented regarding this relationship. For example, two studies evidenced that having only one parent or nonparents as the primary caregiver would decrease the opportunity to seek oral healthcare services, owing to limited time and dental neglect [31,34]. On the other hand, remittances sent home from migrant parents provide greater chance for healthcare utilization in LBC families [35,36]. Grandparents, as the main decision makers, may prefer to invest in children’s health in a convenient healthcare environment [36], or may not invest due to limited education and health beliefs [31].

The education level of caregivers, such as the mother, significantly affects children’s oral health resource utilization, and children with highly educated caregivers have more chances to seek dental care [20,33,37]. In the present study, the low rate of seeking dental care for children was not surprising, as most caregivers did not complete the compulsory nine years of education. This study presents a miniature view of Yunnan, located in southwest China, where the average of education years was 8.82 in 2020 [38]. Improving the education level in undeveloped areas is a long-term and necessary program for improving people’s wellbeing, including healthcare resource utilization.

As expected, a high prevalence of dental neglect exists in LBC families, but was also found in NLBC families in our study. Caregivers tend to be proud of their school-aged children if they exhibit independent living skills, such as independently brushing their teeth [39]. This attitude more obviously exists in parental migration families [39]. However, that childrearing lays a foundation for dental neglect. According to WHO recommendations, children under 8 years old cannot effectively brush their teeth and still need care from their parents [40].

There were no similar patterns in any of the heatmaps that clustered participants’ background characteristics, especially for being left behind or not, in the reasons for seeking or not seeking dental care. This means there was no association between being left behind and reasons for seeking or not seeking dental care. Most reasons for seeking or not seeking dental care chosen by caregivers reflected their oral health knowledge and awareness. Our study showed that children without significant dental pain do not inspire neglectful caregivers to take them to visit the dentist, which is supported by other studies [41,42].

The FDI World Dental Federation stated that “by 2030, essential oral health services [will be] integrated into healthcare in every country, and appropriate quality oral healthcare [will become] available, accessible, and affordable for all” [43]. While this statement must be strongly supported, our study showed that availability of dental services alone is insufficient to guarantee that the children will receive oral health services and have good oral health. People’s lack of awareness on the importance of oral health is one of the final common paths of failure to utilize oral health care. The important implication of our study is that we need to improve awareness of the caregivers of the rural population regardless of whether the child is left behind or not.

This is the first follow-up study to compare the rate of compliance with professional dental advice between rural caregivers of NLBC and LBC. There were limitations in this study. Firstly, there was a short follow-up duration. A longer time may allow for caregivers to respond to the advice with a better outcome. Secondly, about 40% (154) of caregivers in the revisited survey differed from those in the first survey. Nevertheless, those in the first survey transferred the information on their children’s oral health advice to other caregivers in the family.

## 5. Conclusions

Our study found evidence that compliance with dental advice on supervising oral health behaviors differed between caregivers of LBC and NLBC, but compliance with seeking dental care was not different. We demonstrated that SES was associated with knowledge and awareness of the importance of oral health and was directly related to receiving oral health service without mediation through the two former variables. The main reason for not complying with dental care advice among caregivers was the perception of dental problems not being severe and causing no pain to their children. Thus, to promote oral health in this area, caregivers should be educated more about the importance of oral health.

## Figures and Tables

**Table 1:** Comparison of compliance with advice on characteristics of participants, *n* (%).

Characteristic		Supervise the Oral Health Behaviors of the Child			Take the Child to Seek Dental Care		
		No	Yes	*p*	No	Yes	*p*
N		139 (35.7)	250 (64.3)		261 (67.1)	128 (32.9)	
Left behind				0.03			0.70
	NLBC	55 (30.1)	128 (69.9)		121 (66.1)	62 (33.9)	
	LBC	84 (40.8)	122 (59.2)		140 (68.0)	66 (32.0)	
Children factors							
Age of children (years)				0.89			0.63
	6	48 (34.8)	90 (65.2)		92 (66.7)	46 (33.3)	
	7	68 (37.0)	116 (63.0)		127 (69.0)	57 (31.0)	
	8	23 (34.3)	44 (65.7)		42 (62.7)	25 (37.3)	
Sex of children				0.23			0.26
	Female	70 (38.9)	110 (61.1)		126 (70.0)	54 (30.0)	
	Male	69 (33.0)	140 (67.0)		135 (64.6)	74 (35.4)	
Caries in primary and permanent teeth at baseline				0.92			0.10
	Caries free	8 (34.8)	15 (65.2)		19 (82.6)	4 (17.4)	
	Caries	131 (35.8)	235 (64.2)		242 (66.1)	124 (33.9)	
Snacking frequency of children				0.86			0.97
	Few/Never	56 (37.3)	94 (62.7)		100 (66.7)	50 (33.3)	
	Once	29 (34.1)	56 (65.9)		58 (68.2)	27 (31.8)	
	Twice or more	53 (34.9)	99 (65.1)		102 (67.1)	50 (32.9)	
Toothbrushing frequency of children				0.16			0.49
	Not brush every day	27 (39.7)	41 (60.3)		48 (70.6)	20 (29.4)	
	Once	74 (38.7)	117 (61.3)		130 (68.1)	61 (31.9)	
	Twice or more	37 (29.1)	90 (70.9)		80 (63.0)	47 (37.0)	
Medical insurance of children				0.57			>0.99
	No	4 (28.6)	10 (71.4)		10 (71.4)	4 (28.6)	
	Yes	135 (36.0)	240 (64.0)		251 (66.9)	124 (33.1)	
Caregivers’ factors							
Age of caregivers (years)				0.07			0.06
	≤29	20 (38.5)	32 (61.5)		33 (63.5)	19 (36.5)	
	30–40	54 (29.7)	128 (70.3)		114 (62.6)	68 (37.4)	
	41–84	55 (42.0)	76 (58.0)		98 (74.8)	33 (25.2)	
Sex of caregivers				0.74			0.47
	Female	103 (35.3)	189 (64.7)		193 (66.1)	99 (33.9)	
	Male	36 (37.1)	61 (62.9)		68 (70.1)	29 (29.9)	
Education level of caregivers				<0.001			<0.001
	Low	76 (40.6)	111 (59.4)		138 (73.8)	49 (26.2)	
	Medium	54 (38.3)	87 (61.7)		95 (67.4)	46 (32.6)	
	High	9 (14.8)	52 (85.2)		28 (45.9)	33 (54.1)	
Occupations of caregivers				0.02			0.09
	Blue-collar	120 (38.6)	191 (61.4)		215 (69.1)	96 (30.9)	
	White-collar	19 (24.4)	59 (75.6)		46 (59.0)	32 (41.0)	
Oral health awareness of caregivers				0.04			0.06
	Not appropriate	63 (42.0)	87 (58.0)		109 (72.7)	41 (27.3)	
	Appropriate	76 (31.8)	163 (68.2)		152 (63.6)	87 (36.4)	
Oral health knowledge of caregivers				0.46			0.10
	Not appropriate	47 (33.3)	94 (66.7)		102 (72.3)	39 (27.7)	
	Appropriate	92 (37.1)	156 (62.9)		159 (64.1)	89 (35.9)	
Family factors							
Siblings of children				0.23			0.03
	No	38 (31.4)	83 (68.6)		72 (59.5)	49 (40.5)	
	Yes	101 (37.7)	167 (62.3)		189 (70.5)	79 (29.5)	
Family economic status				0.24			0.14
	Income < expense	56 (39.7)	85 (60.3)		101 (71.6)	40 (28.4)	
	Income ≥ expense	83 (33.7)	163 (66.3)		158 (64.2)	88 (35.8)	
Family belongings				0.90			0.05
	Low number	94 (35.6)	170 (64.4)		186 (70.5)	78 (29.5)	
	High number	45 (36.3)	79 (63.7)		75 (60.5)	49 (39.5)	

Note: blue-collar workers—farmers, stay-at-home, others; white-collar workers—business, officials; *p* value by Chi-square test, or Fisher’s exact test.

**Table 2:** Reasons of seeking or not dental care of caregivers of NLBCs and LBCs (multiple answers).

Options		Overall	NLBCs	LBCs	*p*
(A) Reasons for not seeking dental care					
	N	261	121	140	
	Dental disease was not severe	194 (74.3)	90 (74.4)	104 (74.3)	1.00
	Having no pain	164 (62.8)	71 (58.7)	93 (66.4)	0.25
	No time	135 (51.7)	69 (57.0)	66 (47.1)	0.14
	No need to cure primary teeth	76 (29.1)	40 (33.1)	36 (25.7)	0.24
	Responsibility of parents	25 (9.6)	4 (3.3)	21 (15.0)	<0.01
	Inconvenience	15 (5.7)	3 (2.5)	12 (8.6)	0.07
	Economic issue	14 (5.4)	6 (5.0)	8 (5.7)	1.00
	Fear of pain	12 (4.6)	10 (8.3)	2 (1.4)	0.02
	No dentists nearby	9 (3.4)	4 (3.3)	5 (3.6)	1.00
	Self-medication	5 (1.9)	1 (0.8)	4 (2.9)	0.38
	Difficulty of registration	4 (1.5)	3 (2.5)	1 (0.7)	0.34
	Seeking help from experiencers	4 (1.5)	2 (1.7)	2 (1.4)	1.00
	Seeking help from relatives	2 (0.8)	2 (1.7)	0 (0.0)	0.21
(B) Reasons for seeking dental care					
	N	128	62	66	
	Following the advice	106 (88.3)	51 (87.9)	55 (88.7)	1.00
	Oral health is important	77 (64.7)	39 (67.2)	38 (62.3)	0.71
	Reliable dentists	10 (8.3)	2 (3.4)	8 (12.9)	1.00
	Having dentists nearby	9 (7.5)	6 (10.3)	3 (4.8)	0.31
	Can be reimbursed	2 (1.7)	1 (1.7)	1 (1.6)	1.00

## Data Availability

Data is available from the corresponding author for reasonable reasons.

## Supplementary Materials

The following supporting information can be downloaded at: https://www.mdpi.com/article/10.3390/ijerph20021068/s1, Figure S1: Report of oral health examination of the child; Figure S2: Concept framework of structural equation modeling; Figure S3: Heatmap of clustering reasons for not seeking dental care by type of caregiver; Figure S4: Heatmap of clustering reasons for not seeking dental care by education of caregiver; Figure S5: Heatmap of clustering reasons for not seeking dental care by occupation of caregiver; Figure S6: Heatmap of clustering reasons for seeking dental care by type of caregiver; Figure S7: Heatmap of clustering reasons for seeking dental care by education of caregiver; Figure S8: Heatmap of clustering reasons for seeking dental care by occupation of caregiver; Table S1: CFA results on latent variables; Table S2: Goodness of fit measures of the model.
