# Supplementary material for: Pathway of Effects of Socioeconomic Status on Rural Left-behind Children to Receive Oral Health Services: A Structural Equation Modeling
Source: Int J Environ Res Public Health. 2023 Jan 7;20(2):1068. doi: 10.3390/ijerph20021068 (PMC9858901; doi:10.3390/ijerph20021068)
Supplement: Supplementary file 1 [file ijerph-20-01068-s001.zip › ijerph-2121769-supplementary.pdf]

# Pathway of Effects of Socioeconomic Status on Rural Left-behind Children to Receive Oral Health Services: A Structural Equation Modeling

Sichen Liu, Virasakdi Chongsuvivatwong, Shinan Zhang and Angkana Thearmontree

## Figures and Tables Caption List in Supplemental Materials

Figure S1 Report of oral health examination of the child
Figure S2 Concept framework of structural equation modeling
Figure S3 Heatmap of clustering reasons for not seeking dental care by type of caregiver
Figure S4 Heatmap of clustering reasons for not seeking dental care by education of caregiver
Figure S5 Heatmap of clustering reasons for not seeking dental care by occupation of caregiver
Figure S6 Heatmap of clustering reasons for seeking dental care by type of caregiver
Figure S7 Heatmap of clustering reasons for seeking dental care by education of caregiver
Figure S8 Heatmap of clustering reasons for seeking dental care by occupation of caregiver

Table S1 CFA results on latent variables
Table S2 Goodness of fit measures of the model

## Report of oral health examination of (name of the child)

Date of examination ____________
Date of report ____________

Requirement: Please tick "√" in the front of the corresponding option.

To parents of __________ :

After an oral health examination, we found that your child:

- Oral hygiene ☐ Good
  ☐ Fair
  ☐ Poor
- Tooth decay ☐ No obvious cavities
  ☐ At least ________cavities

**We recommend that you:**

**1. Bring the child to see dentist for**

☐ 1.1 Extraction of teeth #____________________
☐ 1.2 Filling of teeth # ____________________
☐ 1.3 Scaling / tooth cleaning
☐ 1.4 Preventive care; Sealant or professional; Fluoride application
☐ 1.5 Others

**2. Supervise or close care on your child oral health behaviours on**

2.1 Toothbrushing

☐ Brush in the morning
☐ Brush before going to bed
☐ Brush with longer time
☐ Others

2.2 Snacking

☐ Less frequent snaking
☐ Limited amount of sweet foods
☐ Others

This examination is a preliminary oral health survey and may not be able to detect cavities and other dental problems that are not clinically apparent. We provide preliminary advice and recommend that you consult with a professional dental practice.

Public Dental Health Department,
School of Stomatology of Kunming Medical University
Day, month, Year

Figure S1. Report of oral health examination of the child.

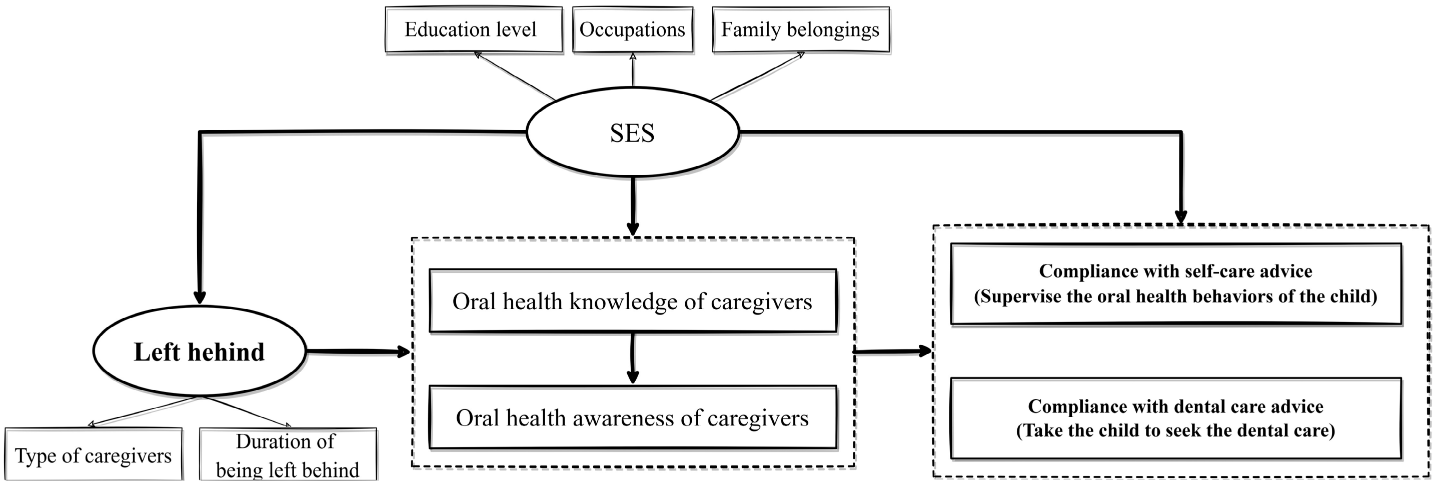


Figure S2. Concept framework of structural equation modeling.

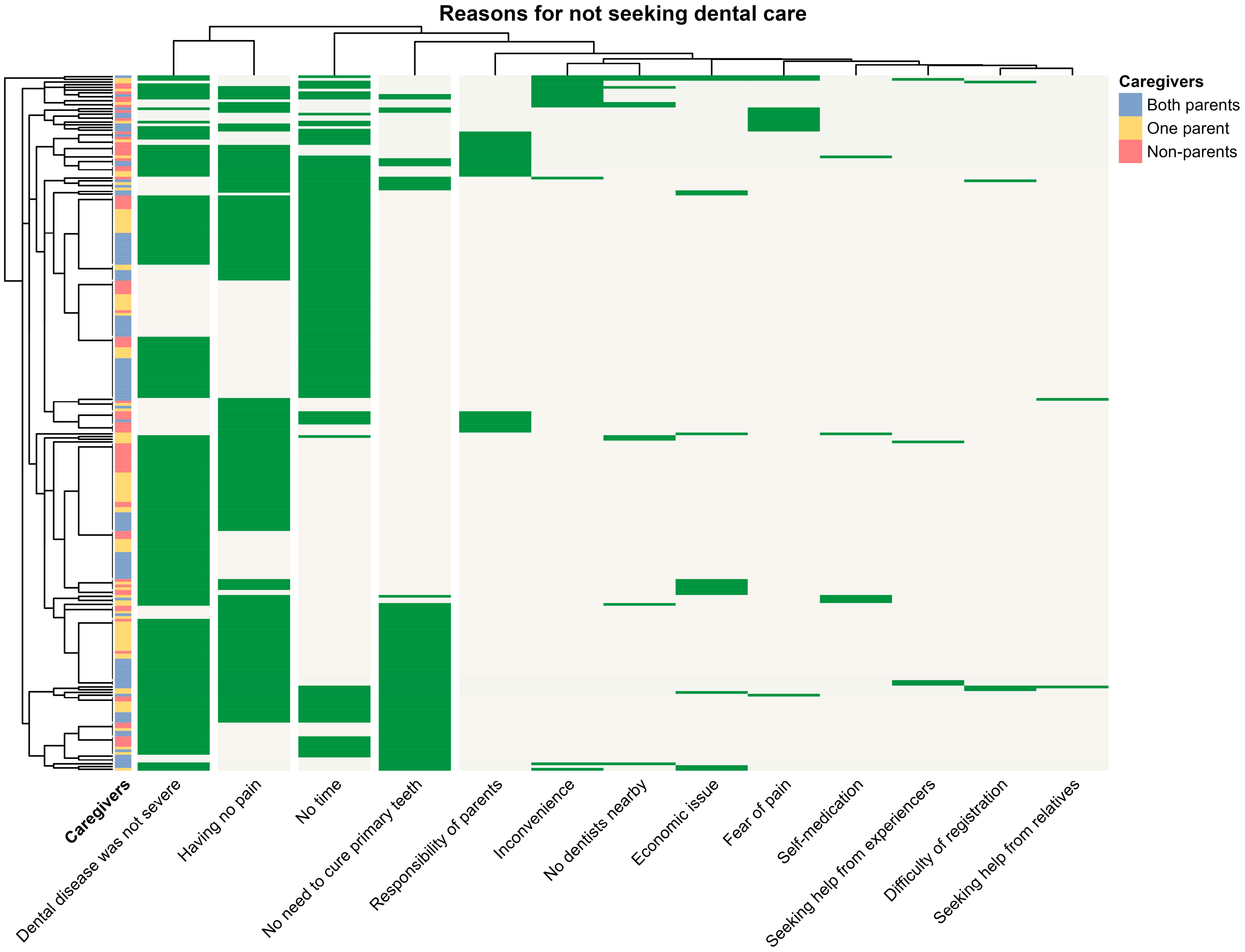


Figure S3. Heatmap of clustering reasons for not seeking dental care by type of caregiver.

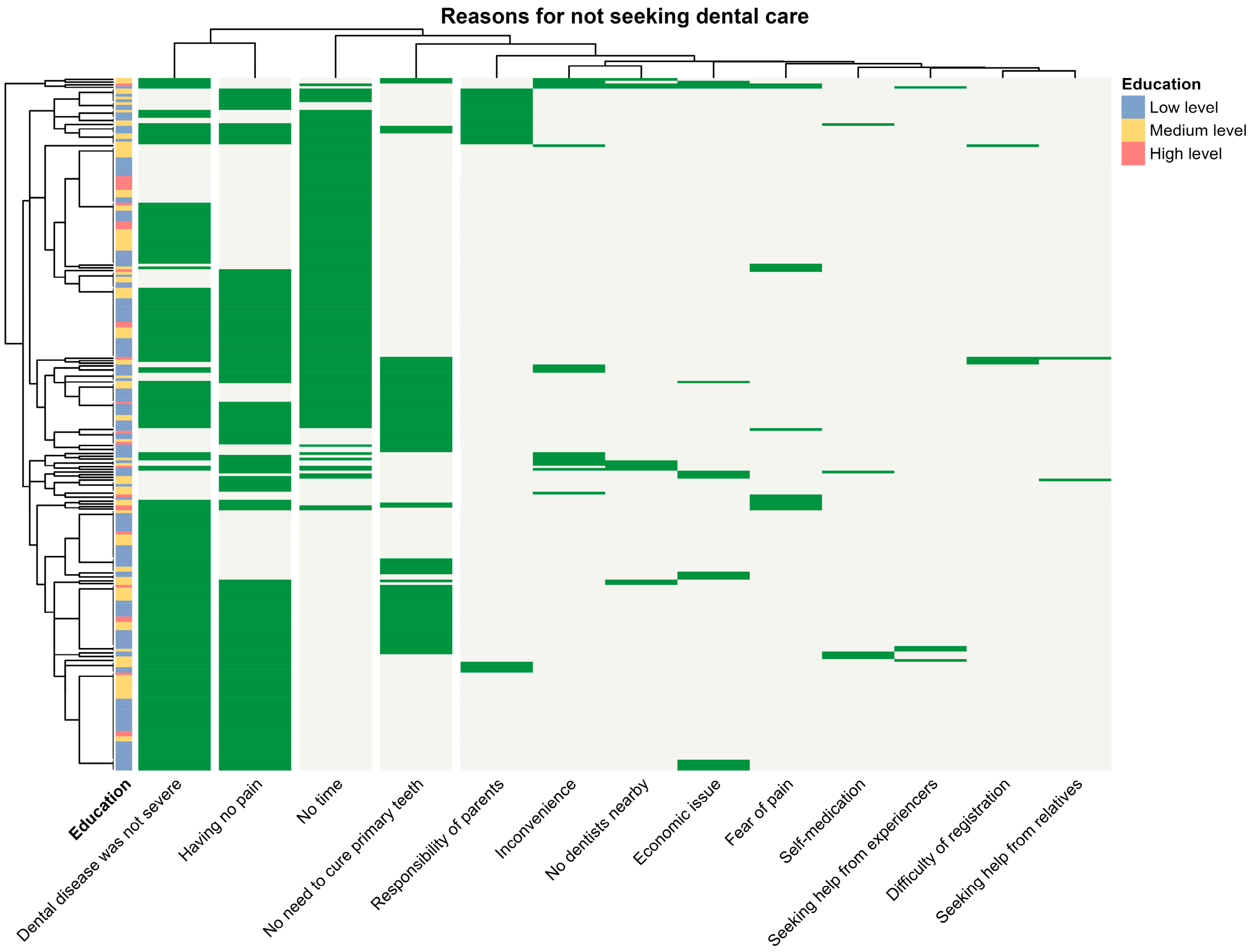


Figure S4. Heatmap of clustering reasons for not seeking dental care by education of caregiver.

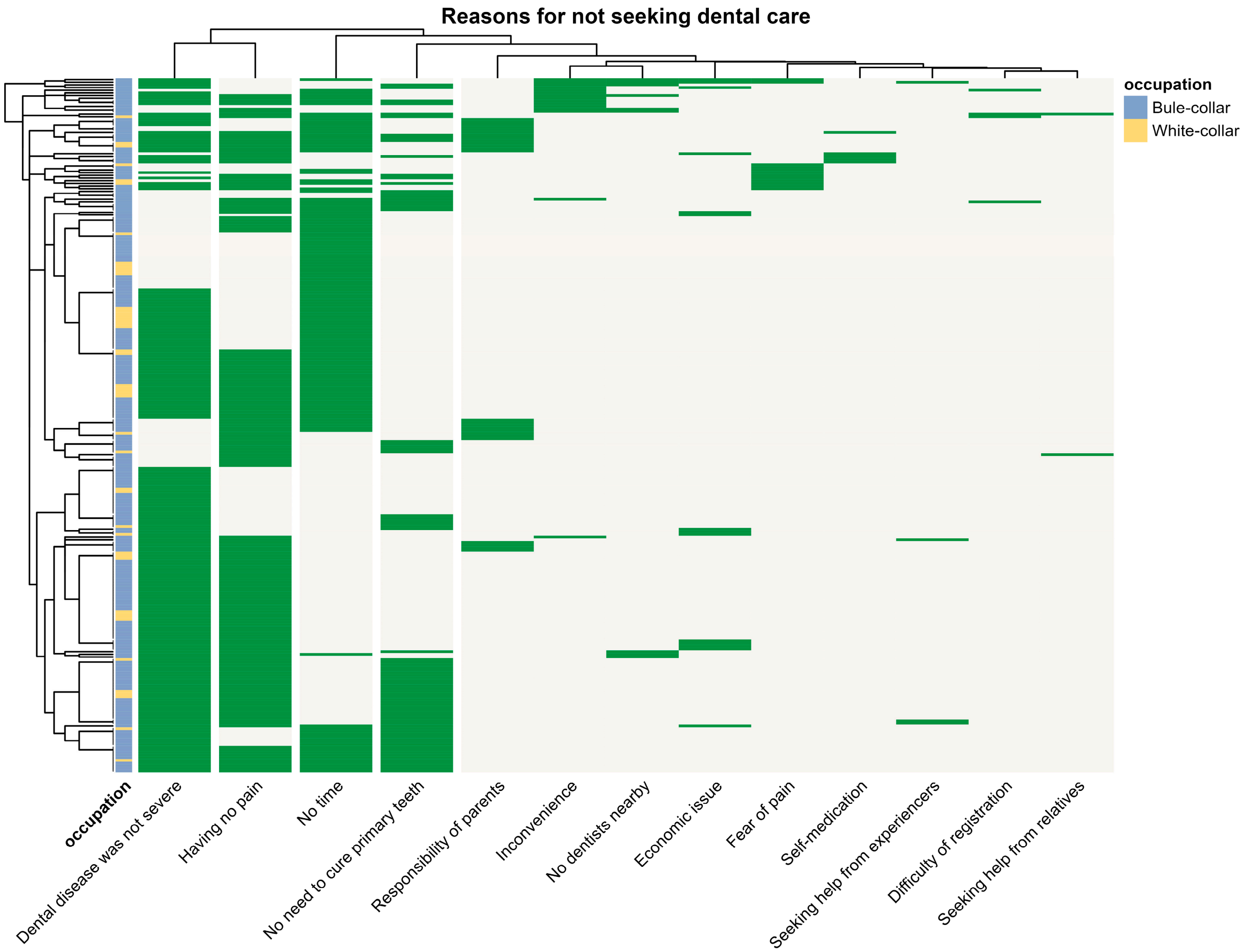


Figure S5. Heatmap of clustering reasons for not seeking dental care by occupation of caregiver.

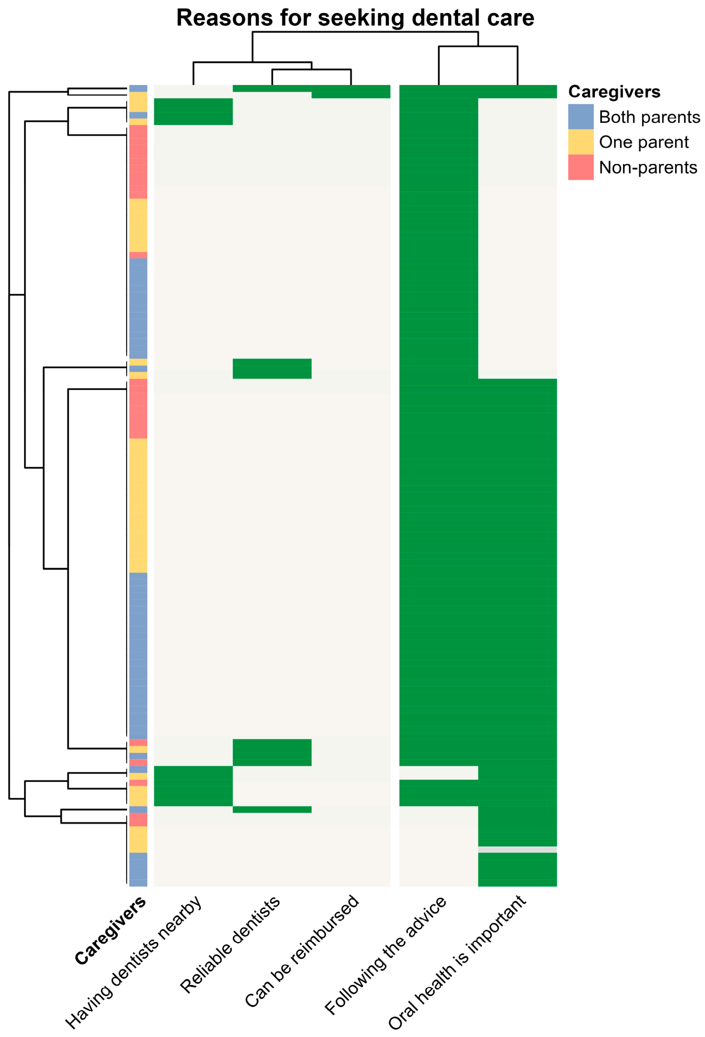


Figure S6. Heatmap of clustering reasons for seeking dental care by type of caregiver.

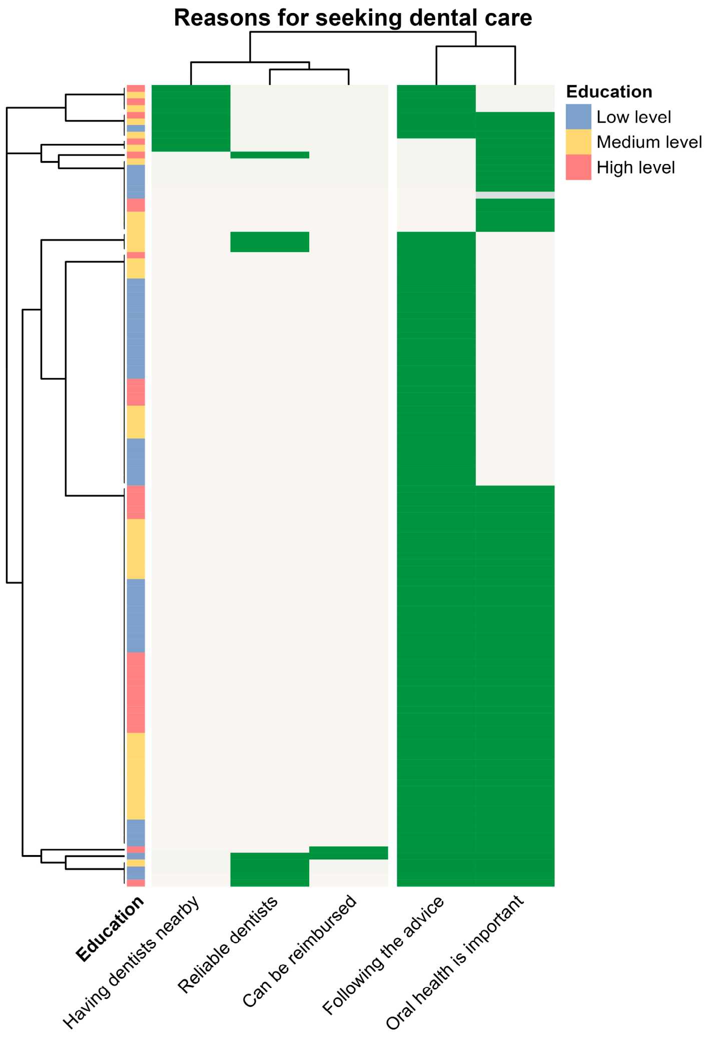


Figure S7. Heatmap of clustering reasons for seeking dental care by education of caregiver.

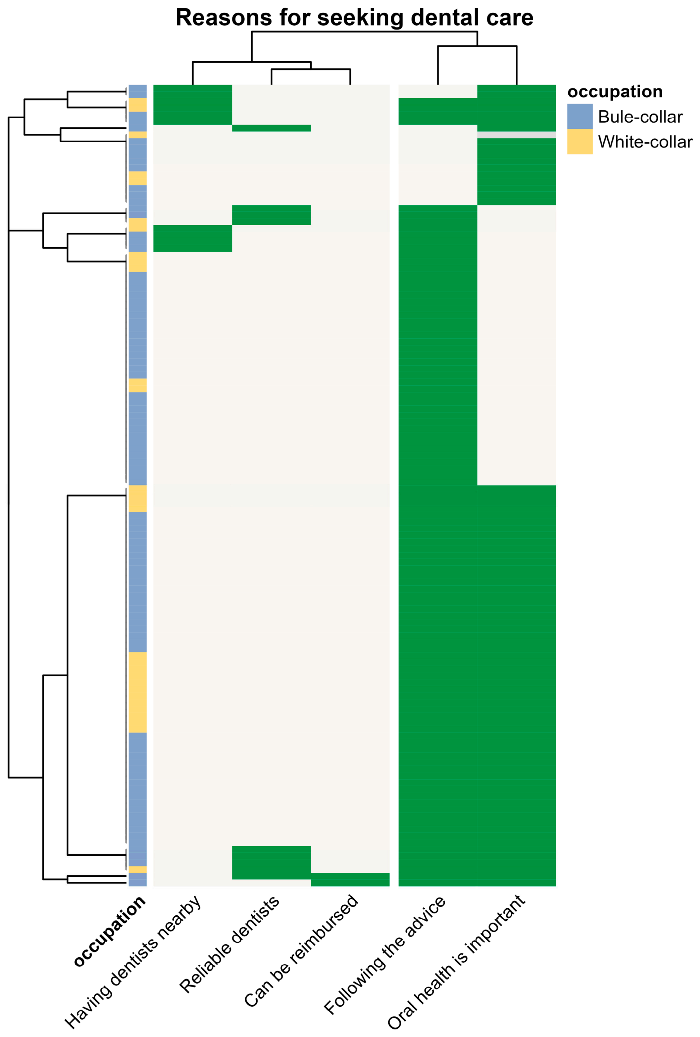


Figure S8. Heatmap of clustering reasons for seeking dental care by occupation of caregiver.

Table S1. CFA results on latent variables.

| Factors | Factor Loading | Internal Consistency Reliability | Convergent Validity |
|---|---|---|---|
| | | Cronbach's alpha | AVE |
| Acceptable level | > 0.40 | > 0.40 | > 0.50 |
| Left behind | | 0.92 | 0.87 |
| Type of caregivers | 0.94 | | |
| Duration of being left behind | 0.93 | | |
| SES | | 0.47 | 0.31 |
| Education level of caregivers | 0.70 | | |
| Occupations of caregivers | 0.46 | | |
| Family belongings | 0.31 | | |

Notes: AVE: Average variance extracted.

Table S2. Goodness of fit measures of the model.

| Fit Index | Recommend Levels | This Model |
|---|---|---|
| $\chi^2$/df | < 5.00 | 0.40 |
| RMSEA | < 0.08 | 0.00 |
| SRMR | < 0.08 | 0.02 |
| GFI | > 0.90 | 0.99 |

$\chi 2$ /df: the chi-squared fit statistic;
RMSEA: root-mean-square error of approximation;
SRMR: standardized root mean square residual;
GFI: goodness-of-fit statistic.
